# Are Maternal Adverse Childhood Experiences Associated with Their Preschool Children’s Sleep Disruptions? Longitudinal Mediation Through Mothers’ Depressive Symptoms and Children’s Screen Time

**DOI:** 10.3390/children13010139

**Published:** 2026-01-18

**Authors:** Stefan Kurbatfinski, Lalith Nandakumar, Janelle Boram Lee, Gerald F. Giesbrecht, Nicole Letourneau

**Affiliations:** 1 Department of Community Health Sciences, Cumming School of Medicine, University of Calgary, Calgary, AB T2N 1N4, Canada; stefan.kurbatfinski@ucalgary.ca (S.K.);; 2 Owerko Centre, Alberta Children’s Hospital Research Institute, Calgary, AB T2N 1N4, Canada; lalith.nandakumar@ucalgary.ca (L.N.);; 3 Data Science and Analytics, Faculty of Sciences, University of Calgary, Calgary, AB T2N 1N4, Canada; 4 Departments of Pediatrics and Community Health Sciences, Cumming School of Medicine, University of Calgary, Calgary, AB T2N 1N4, Canada; 5 Hotchkiss Brain Institute, University of Calgary, Calgary, AB T2N 1N4, Canada; 6 Faculty of Nursing, University of Calgary, Calgary, AB T2N 1N4, Canada; 7 Departments of Pediatrics, Psychiatry, and Community Health Sciences, Cumming School of Medicine, University of Calgary, Calgary, AB T2N 1N4, Canada

**Keywords:** children’s sleep disruptions, maternal adverse childhood experiences, maternal depressive symptoms, screen time, the APrON Study

## Abstract

**Highlights:**

**What are the main findings?**

- Maternal adverse childhood experiences (ACEs) did not exert direct effects on children’s sleep disruptions.
- Maternal depressive symptoms may mediate the association between maternal ACEs and children’s sleep disruptions.

**What is the implication of the main finding?**

- Screening for maternal depressive symptoms when children present with sleep disruptions may improve sleep quality, especially when there is an awareness of maternal exposure to ACEs.
- If depressive symptoms arise from maternal ACEs, interventions targeting depression might be enhanced by focusing on mothers’ early childhood experiences.

**Abstract:**

Background: Children of mothers exposed to adverse childhood experiences (ACEs) may be at increased risk of sleep disruptions, such as night waking, due to potential suboptimal caregiving or living conditions. Mothers’ ACEs are also associated with maternal depressive symptoms, which in turn are associated with children’s screen time and sleep disruptions, revealing relevant, but unexplored, mediation pathways. This Canadian study investigated if mothers’ ACEs were associated with their 5-year-old children’s sleep disruptions (1) directly and (2) indirectly through independent or serial mediation via maternal depressive symptoms and/or children’s screen time. Methods: Data (*n* = 622; maternal mean age 32.3 years, 88.4% white) came from the longitudinal APrON Study. ACEs were measured 1 year postpartum. Mother’s depressive symptoms were measured across prenatal and postnatal timepoints. Children’s evening screen time (i.e., number of days in a week children engaged in one hour of screen time before bedtime) and sleep disruptions (number of days in a week their child wakes up multiple times) were measured at 5 years postpartum using adapted scales (52.9% male). PROCESS was used to assess for mediation. Results: Mothers’ ACEs had an indirect effect on their children’s sleep disruptions through mothers’ mean depressive symptoms (effect = 0.018, 95% CI [0.006, 0.034]), but not through children’s screen time. No other effects (i.e., direct, total) were observed. Conclusions: Although replication studies are warranted, this novel study reveals that the effects of maternal ACEs on children’s sleep disruptions may operate indirectly with effects potentiated through maternal depressive symptoms, thus serving as a target for intervention.

## 1. Introduction

Children’s sleep disruptions, defined in this study as the number of times a child wakes up throughout their sleep, serve as a proxy for sleep quality [1]. Sufficient sleep allows the brain to process daily events, consolidate memories, and foster the development and refinement of neural pathways that can affects one’s emotions, thoughts, and behaviours [2]. In young children, sleep disruptions have been associated with suboptimal cognitive function [3]. While sleep disruptions are common in infants and toddlers, they tend to decrease around ages 4 to 5 [4]. Therefore, frequent sleep disruptions among preschoolers may be reflective of other underlying concerns, such as emotional problems or stress dysregulation [4]. Indeed, improvements in children’s sleep disruptions and overall sleep quality have been associated with better intellectual functioning and behavioural outcomes [5], among other benefits, serving as an important measure of children’s health.

Although various parental factors are associated with children’s sleep disruptions (e.g., parental presence at bedtime) [6], maternal adverse childhood experiences (ACEs) may also serve as an important, yet understudied, predictor variable. Defined as adverse events experienced before 18 years of age (e.g., abuse, neglect, parental concerns such as mental health concerns, parental loss and conflict) [7], ACEs, especially those of mothers, can intergenerationally affect their children’s sleep disruptions both directly (e.g., suboptimal mental health) and indirectly (e.g., via fetal programming mechanisms) [8]. Preschool children are an especially important group to examine since this developmental stage is marked by rapid changes in neurodevelopment [9,10]. This neuroplastic period increases children’s sensitivity to their environmental conditions [11], whereby ACEs can negatively affect development and result in worsened outcomes [12], such as sleep disruptions.

**Figure 1:**
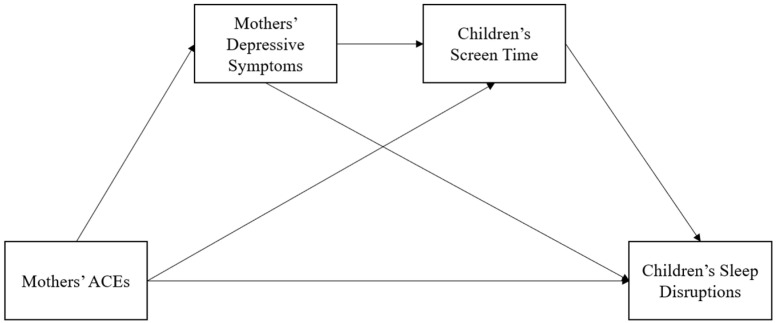
Proposed mediation model.

In addition to examining the association between maternal ACEs and children’s sleep disruptions, investigating potential mediators of the association to understand the transmission of effects is warranted. Maternal ACEs are strongly associated with depressive symptoms across the lifespan [13]. In turn, maternal depressive symptoms can influence children’s bedtime routines and cognitive function in ways which contribute to sleep disruptions [14]. Children may also adopt maladaptive behaviours as ways of regulating vis-à-vis their mothers’ ACEs and/or depressive symptoms [15,16], such as screen time [17,18], potentiating sleep disruptions [19]. So, mediation of the association via maternal depressive symptoms and/or children’s screen time (i.e., independent mediation or serial) is theoretically plausible, but not yet investigated, beckoning exploration (Figure 1).

### 1.1. Parental ACEs and Their Link to Children’s Sleep Quality

Parents who have experienced ACEs often report numerous adverse health outcomes [20] which can directly or indirectly impact their children’s sleep quality [21]. Adults exposed to 4 or more ACEs have 4.7-, 3.7-, and 37.5-times higher odds of experiencing depression, anxiety, or suicidal ideation, respectively, compared to adults with no ACEs. They also face greater odds of various physical health conditions [20], all of which can undermine the quality of care that they provide to their children [22,23,24]. Children of mothers exposed to ACEs are also more likely to experience ACEs themselves, reflecting the intergenerational continuity of parenting phenomenon [25]. Direct exposure to ACEs may heighten children’s levels of stress [25,26] which can exacerbate their risk of experiencing sleep disruptions [27]. Taken together, maternal ACEs serve as a probable but understudied correlate of children’s sleep disruptions.

While researchers have examined the association between maternal/parental ACEs and sleep problems in children (e.g., sleep talking, insomnia) [28,29,30,31], none have examined sleep disruptions as the primary outcome. However, Ciciolla et al. did reveal a significant correlation between mothers’ ACEs and their 6-week-old infant’s sleep difficulties (which included number of night time awakenings) [31]. Nevertheless, no direct effects were explored, or at least reported, via mediation analysis [31]. Although theoretical models support the plausibility of maternal ACEs exacerbating children’s sleep disruptions, current evidence remains limited.

### 1.2. Maternal ACEs and Parental Depressive Symptoms

Depressive symptoms, such as fatigue, withdrawal, and unresponsiveness, are characterized by changes in behaviour and mood that interfere with daily living and interest in activities [32]. A plethora of research has supported strong, positive associations between one’s exposure to ACEs and the emergence of depressive symptoms in adulthood [13]. In fact, a meta-analysis of 13 studies revealed a 4.74-times higher odds of depression among adults exposed to four or more ACEs compared to adults with none after removing outliers [13]. The positive association between ACE exposure and depressive symptoms can be attributed to various reasons, including, but not limited to, dysregulated stress response systems, epigenetic mechanisms, altered cognition, and inflammation, all of which are reflective of chronic stress [8].

Depressive symptoms among mothers are known to undermine children’s sleep quality by negatively affecting their stress response systems [14,33,34]. While not focused specifically on sleep disruptions, a meta-analysis revealed 1.82- and 1.65-times higher odds of children experiencing sleep problems in the early childhood period if they were exposed to maternal prenatal or postnatal depression, respectively [35]. Since maternal ACEs are associated with maternal depressive symptoms [13], which in turn are associated with children’s sleep problems [14,33,34], maternal depressive symptoms could mediate the association between maternal ACEs and children’s sleep disruptions.

Only one study has explored the mediation effect of parental depressive symptoms with children’s sleep problems more broadly as the outcome, but it did not support an indirect effect [28]. Also, this study focused on fathers’ ACEs and depressive symptoms as opposed to mothers’ [36]. This could have lent to the null indirect effect since studies consistently emphasize that mothers’ ACEs [37] and depressive symptoms [38,39] more strongly impact their children’s outcomes, likely an embodiment of mothers often assuming primary parenting roles due to ongoing, socially constructed gender norms [36]. Therefore, a gap in research persists: examination of the mediation of the association between maternal ACEs and preschool children’s sleep disruptions via mothers’ depressive symptoms.

### 1.3. Children’s Screen Time: A Plausible Correlate of Children’s Sleep Disruptions

Maternal ACEs have been modestly associated with children’s screen time, likely as a way for children to self-regulate or for parents to manage their children during dysregulation [15,16]. Consequently, children’s screen time, especially before bedtime [40], has been strongly linked to poorer sleep quality among children [41]. However, when specifically examining sleep disruptions as the outcome, one cross-sectional study showed an increased odds of sleep disruptions among children engaging in higher amounts of screen time [42] whereas another reported no association between touchscreen use and sleep disruptions [43]. On the other hand, one randomized-controlled trial revealed that the removal of screen use before bedtime was associated with fewer nighttime awakenings among toddlers [44]. Thus, the limited empirical evidence demonstrates mixed findings [42,43,44].

Various mechanisms can help explain the effect of screen time on sleep disruptions, including light emissions from screens and physical or mental arousal [19,40,43]. Specifically, blue light emissions, ranging between 400 and 500 nanometers [45], are most harmful to sleep as they stimulate the brain and can alter the circadian rhythm (e.g., affect melatonin production) [40]. Content is also relevant; games or videos which induce excitement and stimulate the sympathetic system (i.e., violent games) can result in psychological arousal and therefore alter sleep behaviours and induce sleep disruptions [43]. These behavioural and physiological changes may be particularly relevant among mothers exposed to ACEs and their children. To prevent negative impacts, the Canadian Paediatric Society has recommended that parents limit young children’s screen time to one hour a day [46].

To date, only one study has explored the association between mothers’ ACEs and their children’s screen time using a Chinese sample, reporting a positive association [47]. Pronounced screen time in children can emerge when mothers are less responsive [48], which is more common among children of mothers exposed to ACEs and who experience depressive symptoms [49]. Regardless, since maternal ACEs are associated with children’s screentime [15,16], and children’s screentime may be associated with their sleep disruptions [42,44], children’s screen time may serve as another probable mediator of the association.

### 1.4. Potential Serial Mediation

Investigating whether the association between mothers’ ACEs and their preschool children’s sleep disruptions is mediated via mothers’ depressive symptoms and children’s screen time through a serial model also warrants further attention. While maternal ACEs are strongly associated with higher levels of depressive symptoms both prenatally [50] and postnatally [13], maternal depressive symptoms are also correlated with children’s screen time as children may turn to screen time to self-regulate if their mothers are less responsive [17,51]. Depressed mothers may also engage in increased screen time themselves, a prominent predictor of their own children’s screen time [52]. Therefore, these robust individual pathways collectively point to a promising serial mediation model: mothers’ ACEs may first exacerbate their depressive symptoms, which then increase their children’s screen time, ultimately exacerbating the occurrence of sleep disruptions. Despite strong theoretical support for this model, no studies have yet examined this indirect effect. Examining this additional mediation model can help to clarify mechanisms and more optimally inform interventions targeting mother–child dyads in which mothers report ACE exposure or depressive symptoms.

### 1.5. Relevant Covariates of Interest

Numerous variables have also been linked to children’s sleep disruptions. Socioeconomic factors, including ethnicity, household income, and maternal educational attainment can influence parental presence during bedtime and living conditions (e.g., nutritional intake, household function), affecting children’s sleep [53]. Birth outcomes, including gestational age at delivery and birthweight are also strongly linked to children’s brain development, potentially influencing brain regions related to sleep behaviours [53]. Sleep can also differ based on child sex-assigned-at-birth since environmental conditions can differentially impact children’s sleep based on female versus male biological differences. Including these variables as confounders is critical to control for extraneous effects.

### 1.6. Purpose of the Study

This appears to be the first study examining whether mothers’ ACEs are associated with their children’s sleep disruptions: (1) directly; (2) indirectly through mothers’ depressive symptoms or children’s screen time (defined as the number of days their child engaged in screen time for one hour before bedtime); and (3) indirectly through serial mediation via mothers’ depressive symptoms followed by children’s screen time. We did not expect maternal ACEs to have direct effects on their children’s sleep disruptions. However, we hypothesized that mothers’ ACEs would positively predict children’s sleep disruptions through mothers’ depressive symptoms and children’s screen time, separately, but also via serial mediation whereby (1) maternal ACEs are positively associated with maternal depressive symptoms, (2) maternal depressive symptoms are positively associated with children’s screen time, and (3) children’s screen time is positively associated with their sleep disruptions.

## 2. Methods

### 2.1. Study Design

This longitudinal study used data from the prospective APrON Study located in Alberta, Canada, whereby additional information is available elsewhere [54]. Ethical approval was obtained from the University of Calgary Conjoint Health Research Ethics Board (REB14-1702) and the University of Alberta Health Research Ethics Biomedical Panel (Pro00002954). Informed consent was provided by all participants at the time of enrollment. Re-assent was acquired at each follow-up.

### 2.2. Sample and Inclusion Criteria

Mothers could partake in the APrON Study if they lived in or near Calgary or Edmonton and were able to attend visits at the University of Calgary or the University of Alberta [54]. Other requirements included being at least 16 years of age, able to speak and read English, less than 27 weeks pregnant upon entry, the biological parent of the child, and planning to remain in the area for a minimum of three months postpartum [54]. If mothers could not speak English and did not plan to remain in the area until 3 months postpartum, they were excluded from the study. Data were collected at every trimester of pregnancy and at 3, 6, 12, 24, 36, and 60 months postpartum. Enrolment occurred from 2009 to June 2012, with 5-year follow-up completed roughly around 2017. Of the 1236 mothers who reported on their ACE exposure when children were 12 months old, only 622 completed follow-up on their children’s sleep disruptions at 5 years of age. Therefore, the final analytic sample after imputation was 622 participants. This study’s dataset is not publicly released, but it is available upon reasonable request to the corresponding author.

### 2.3. Measures

#### 2.3.1. Predictor

Maternal ACEs were assessed at approximately 12 months of age with the 10-item, “yes” or “no”, Adverse Childhood Experiences (ACEs) Questionnaire [55]. The ACEs Questionnaire has been employed widely to assess early childhood adversity, despite its modest psychometric validation [56]. Despite its limitations, it consistently demonstrates strong and positive correlations between higher ACE scores and an increased risk for various health outcomes, with dose–response relationships [13].

#### 2.3.2. Outcome

Children’s sleep disruptions were reported by their parents via direct observation at approximately 5 years of age in the form of frequency of nighttime waking. One item developed by the APrON team was used to proxy sleep disruptions over a one-week period: (1) “my child wakes up more than once during the night”. The following 5-point Likert scale was used as possible responses and coded as an ordinal variable ranging from 1 to 5: 1 = “0 days (Never)”, 2 = “1 day (Rarely)”, 3 = “2–4 days (Sometimes)”, 4 = “5–6 days (Usually)”, and 5 = “7 days (Always)”.

#### 2.3.3. Mediators

Maternal Depressive Symptoms. Maternal depressive symptoms were assessed at each trimester of pregnancy and at 3, 6, and 12 months of child age using the Edinburgh Postnatal Depression Scale (EPDS) [57]. The EPDS is a 10-item self-report measure that is well used and validated [57,58,59], with scores ranging from 0 to 30; the higher the score, the greater the level of depressive symptoms. It is used extensively in research to assess for mothers’ depressive symptoms during pregnancy and the early years that follow [57,58,59]. Available data for depressive symptoms across the timepoints were averaged to yield a mean score of mothers’ depressive symptoms, helping to capitalize on repeated data.

Children’s Screen Time. Children’s screen time was measured at 5 years of child age using a single-item question developed by this team: “In the past week, how many days did your child engage in screen-based activities (TV, smartphone, computer, tablet) during the hour before going to bed?”. Possible responses ranged from 0 days to 7 days, with higher values reflecting a greater number of days in which children engaged in screen time for at least 1 h a day before bed.

#### 2.3.4. Covariates

Covariates (i.e., maternal ethnicity, overall household income for the year, highest level of maternal education completed, gestational age at delivery, birthweight, and child sex-assigned-at-birth) were included due to their theoretical importance, not statistical importance. Maternal income, education, and ethnicity were determined at study enrollment through self-report from mothers, while gestational age and birth weight were derived from birth records. Child sex-assigned-at-birth was obtained via parent report and verified by birth record. These variables were selected because they have been shown to be associated with child health and developmental status [53].

### 2.4. Data Imputation

Missing data for most of the variables were less than 10% (Table 1), except for ACEs (13%) and screen time (18%). Little’s test suggested that the data for the entire dataset were missing completely at random (*p* = 0.24). Therefore, data were imputed using multivariate imputation by chained equations (MICE) via the “mice” package in R (Version 3.18.0), a suitable approach for handling data missing completely at random [60]. The process generated 20 complete datasets using the predictive mean matching method. The algorithm was run for 50 iterations to ensure stability of the imputed values. For reproducibility, a random seed was set to 500. Because PROCESS cannot pool results across multiply imputed datasets, we selected one imputed dataset at random for the analyses. However, a sensitivity check was completed by repeating the analysis with three additional randomly selected imputed datasets. Results were consistent in significance, direction, and magnitude, suggesting that the primary findings were not dependent on the choice of imputed dataset.

### 2.5. Data Analysis

All statistical analyses were conducted using R (version 4.4.1). We performed a serial mediation analysis using the PROCESS macro (v5) [61] for R to examine the direct and indirect pathways linking mothers’ ACEs to their children’s sleep disruptions using two sequential mediators, whereby the predictor variable was maternal ACEs, the mediators were maternal depressive symptoms and children’s screen time, and the outcome was children’s sleep disruptions. Covariates included maternal ethnicity, maternal education, household income, gestational age at birth, child birthweight, and child sex-assigned-at-birth. To account for potential measurement error in our self-report measures [61], the analysis incorporated conservative McDonald’s Omega (PROCESS v5) reliability estimates for all the predictor variables. The reliability for the predictor (ACEs) was set to 0.70, and the reliabilities for the mediators (depressive symptoms and screen time) were set to 0.87 and 1.00, respectively. All covariates were set to 1.00. Screen time and covariates were defined as 1.00 because they were single-item questionnaires or birth record data collected on sociodemographic information or birth outcomes that are believed to be accurate. Total effects were used to examine the association between maternal ACEs and children’s sleep disruptions. The significance of the individual and total indirect effects was evaluated using bootstrapping with 5000 resamples to generate bias-corrected 95% confidence intervals (CIs). An indirect effect was considered statistically significant if its 95% CI did not include zero. A fixed seed of 42 was used for the bootstrap estimation.

## 3. Results

### 3.1. Participant Information

Demographic information for the sample (*n* = 622) is provided (Table 1). Main study variables are also descriptively characterized (Table 2). Most mothers self-identified their ethnicity as white, held an undergraduate university degree, and reported a high annual household income (i.e., above CAD 100,000). Children’s sex-assigned-a-birth was approximately evenly distributed. Also, children’s mean gestational age and birthweight fell within normal ranges. Most mothers reported experiencing a low number of ACEs and subclinical depressive symptoms. On average, children engaged in at least one hour of screen time before bedtime for 3.7 days a week.

### 3.2. Direct and Indirect Effects

Controlling for all relevant covariates, the direct effect of maternal ACEs on children’s sleep disruptions was not significant (effect = 0.004, 95% CI [−0.049, 0.056]), nor was the total effect (effect = 0.016, 95% CI [−0.035, 0.067]). However, there was a significant indirect effect of maternal ACEs on children’s sleep disruptions via mothers’ average depressive symptoms (effect = 0.011, 95% CI [0.001, 0.024]), controlling for all relevant covariates. No indirect effect via screen time was supported (effect = 0.002, 95% CI [–0.002, 0.001]). Overall, a total indirect effect of maternal ACEs on children’s sleep disruptions was supported (effect = 0.013, 95% CI [0.001, 0.028]).

### 3.3. Serial Mediation

Controlling for all relevant covariates, the serial indirect effect of maternal ACEs on children’s sleep disruptions via maternal depressive symptoms followed by children’s screen time was not supported (effect = 0.002, 95% CI [–0.002, 0.009]).

## 4. Discussion

This novel study investigated the association between mothers’ ACEs and their preschool children’s sleep disruptions, with findings supporting the hypothesis that ACEs would not be associated with their children’s sleep disruptions. Also, the study examined mediation of the association via mothers’ depressive symptoms and their children’s screen time, with an indirect effect only supported through mothers’ depressive symptoms. Mediation via mothers’ depressive symptoms partially supported our hypothesis although indirect effects were also expected to occur via children’s screen time. Consistent with literature [31,62], findings suggest that the effects of maternal ACEs on children’s sleep disruptions likely operate through indirect pathways, with effects potentiated by maternal depressive symptoms. They also indicate that children’s screen time does not explain the effects of mothers’ ACEs on their children’s sleep disruptions, at least beyond those of maternal mental health symptoms. Although maternal ACEs may directly or indirectly impact children’s sleep quality, few studies have explored this, and even fewer have examined potential mediators of the association, reflecting the importance of our study.

### 4.1. Mothers’ ACEs and Their Preschool Children’s Sleep Disruptions

In accordance with our hypothesis, maternal ACEs were not associated with preschool children’s sleep disruptions; however, the lack of evidence on this association specifically limits comparisons. It is posited that the effects of mothers’ ACEs on their preschool children’s sleep disruptions could be exerted prenatally and/or postnatally [8]. Maternal ACEs have been positively linked to increased stress during pregnancy, resulting in abnormal levels of stress hormones such as cortisol and inflammatory markers, which can impact fetal development [63]. For example, children may experience altered physiological development [64], potentially undermining sleep quality [65]. Also, children born preterm or at lower-than-expected birthweight are less likely to have reached full developmental maturation during pregnancy [8,66], increasing their risk of sleep complications; however, mean values and standard deviations for gestational age and birthweight were well within normal ranges, making this mechanism less probable. Indeed, positive associations between maternal prenatal stress, which is more likely among mothers experiencing ACEs, and children’s sleep disruptions have been observed [67]. Postnatally, mothers may engage in suboptimal interactions with their children [49], which are linked to a number of negative outcomes among children that can result in sleep disruptions [68,69]. Children receiving less optimal interactions with their mothers are more likely to remain in states of stress dysregulation [70,71,72], a major indicator of suboptimal sleep quality [27]. Maternal ACEs are also linked to lower socioeconomic attainment, such as financial difficulties and lower educational attainment [73], potentially impeding in mothers’ ability of providing adequate resources to their child (e.g., appropriate nutritional intake) [74] that may optimize their children’s sleep quality.

In more extreme cases, maternal ACEs may also result in the perpetuation of ACEs, such as children experiencing abuse or neglect, residing with a mother with mental health or substance use conditions, or witnessing family violence [25]. In turn, exposure to ACEs has been shown to increase children’s risk of suboptimal sleep by 1.37 times compared to unexposed children [75]. A systematic review of 73 studies further confirmed robust associations between experiencing childhood maltreatment and the emergence of sleep disturbances [76]. However, none of the studies focused specifically on sleep disruptions among children at 5 years of age [76]. Given that the preschool period is marked by rapid brain development, this study contributes unique findings regarding the impacts of maternal ACEs on preschool children’s sleep disruptions. Collectively, there is apparent potential for maternal ACEs to exacerbate children’s sleep disruptions, but this is more likely to occur indirectly through various pathways.

### 4.2. Mothers’ Depressive Symptoms Serve as a Mediator

A positive, indirect effect of mothers’ ACEs on their preschool children’s sleep disruptions via maternal depressive symptoms was supported, as hypothesized. The observed indirect effect is underpinned by theoretical understandings of how mothers’ prenatal and postnatal depressive symptoms influence children’s sleep quality in early childhood [35]. While one study has examined psychological distress (as a composite of anxiety and depression) as a mediator, the study focused on both parents’ ACEs during the pandemic period and in children aged 6 to 18 years and did not focus on sleep disruptions specifically [62]. Another study focused on the effects of paternal depressive symptoms as opposed to maternal on 3-year-old children’s sleep problems [28]. Therefore, ours is the first study to specifically examine the role of maternal ACEs and depressive symptoms alone (measured via a highly reliable and valid tool) in relation to children’s sleep disruptions outside of the pandemic period, serving as a more baseline analysis focused on mothers who often assume the primary parenting role responsibilities.

Mothers experiencing depressive symptoms during pregnancy are at greater risk of having irregular levels of cortisol or inflammatory markers, both of which can affect fetal development and potentially impair sleep quality later in life [63]. In fact, studies show that children born to mothers with prenatal depression exhibit altered volumes in certain brain regions affiliated with sleep [77], such as the hippocampus and amygdala [78], illustrating how mothers’ ACEs may indirectly impact their children’s sleep quality through prenatal depressive symptoms. Postnatally, mothers’ depressive symptoms vis-à-vis their ACEs, which are linked to fatigue, unresponsiveness, withdrawal, and neglect [32], can result in suboptimal or no response to their children [79], potentially leaving them in states of stress dysregulation [70,71,72]. Whether intentional or not, the consequent high levels of stress and dysregulation during bedtime can result in increased difficulty with children falling and remaining asleep [27]. Our findings, in conjunction with other literature and theoretical understandings [14,27,33,34,65,78], allude to maternal depressive symptoms as an important mediator of the association between mothers’ ACEs and their preschool children’s sleep disruptions.

### 4.3. Null Findings Vis-à-Vis Independent and Serial Mediation Through Children’s Screen Time

Opposite to what was hypothesized, children’s screen time did not independently mediate the association between mothers’ ACEs and their preschool children’s sleep disruptions. Children’s screen time, especially before bed, has been found to be a robust predictor of children’s sleep quality more broadly [19,80], and less so with children’s sleep disruptions [42,43,44], although differences emerge based on the length of exposure and type of device used. One study, conducted in China, examined the association between mothers’ ACEs and their preschool children’s screen time, observing a positive association [47]. However, this study only conducted analyses that provide us with an inference of the initial path of mediation under investigation in our study (i.e., maternal ACEs to children’s screen time). Beyond cultural, linguistic, and sociopolitical differences between China and Canada (the setting of our study), which could impact children’s screen time [81,82], the cited study was conducted in the middle of 2021 when Chinese COVID-19 regulations were in place and strict [83]. This means that the presence of numerous confounding factors could have impacted children’s screen time. In fact, screen time increased among 76.9% students in one Chinese study, with 44.6% engaging in at least 5 h of screen time [84]. Nevertheless, while it was posited that mothers’ ACEs would positively associate with their children’s screen time, and in turn, children’s sleep disruptions, no other mediation studies have been conducted to make the comparisons. It is possible that screen time simply does not account for the link between maternal ACEs and sleep issues in preschoolers within a Western context. However, since this study is the first to employ this mediation model and because our measure of screen time embodies some limitations (see limitations section), this mediation model warrants replication in additional studies before drawing stronger inferences.

What was perhaps more surprising was that children’s screen time did not mediate the association between mothers’ ACEs and their preschool children’s sleep disruptions via serial mediation pathway predicted by maternal depressive symptoms. This is especially notable since, in our study, mothers’ depressive symptoms were predicted by maternal ACEs and have been positively associated with their children’s screen time in other research [14,17,85,86]. Most mothers in the sample self-reported low scores on the EPDS, indicating low levels of depressive symptoms. While non-clinical levels of depressive symptoms in mothers have shown to predict children’s sleep quality [87,88,89], as supported in our findings, they may be less predictive of children’s screen time [17,85,86]. Nevertheless, further studies specifically examining the effects of low, moderate, and high depressive symptoms are necessary to better understand this.

### 4.4. Implications for Research and Clinical Practice

Findings from this study reinforce that maternal ACEs may not exacerbate children’s sleep disruptions directly but instead operate indirectly via maternal depressive symptoms, with no evidence of an indirect effect via children’s screen time. Clinicians and healthcare providers might therefore screen for maternal depressive symptoms when children present with sleep disruptions, especially when there is an awareness of maternal exposure to ACEs. If depressive symptoms are linked to ACE exposure, interventions targeting maternal depression (e.g., eye movement desensitization and reprocessing) could home in on mothers’ early childhood experiences to reprocess early, distressing memories and reduce their impact on mental health. Maternal depression may also compromise parent–child interaction quality; accordingly, short-term interventions could focus on equipping mothers with practical skills and tools to support more nurturant parent–child interactions.

In addition to much needed replication studies, researchers could delve into specific depressive symptoms (e.g., unresponsiveness, fatigue, loss of interest in previously enjoyed activities, irritability) that might be most strongly linked to children’s sleep disruptions to better understand the indirect association between maternal ACEs and children’s sleep disruptions via maternal depressive symptoms. Such research could help develop or refine interventions to target the most relevant depression-related behaviours and prevent impacts on children’s sleep quality. Additional research examining different proxies of screen time and use is necessary to better understand the impact of technology on children’s sleep quality in the context of maternal ACEs and depressive symptoms.

### 4.5. Limitations and Strengths

This study, composed mainly of white mothers, constituted a relatively low-risk sample, as most mothers reported low numbers of ACEs and depressive symptoms, along with high educational attainment and total annual household income. Although the results could differ if the sample distribution was different (e.g., higher proportion of racialized mothers), findings are still generalizable to populations with similar demographics. Screen time was measured as the number of days a week that children used screens for one hour before bedtime; this measure does not account for the possible differential impacts that can be derived from using different devices, accessing diverse content, and the overall amount of time children expend in screen time. However, the cut-off of one hour aligns with public health recommendations targeting young children as set forth by the Canadian Paediatric Society [46]. Some important underlying conditions that are correlated with sleep disruptions were not included in the analysis as potential explanatory variables, including behavioural insomnia, sleep disordered breathing, restless legs, and other pervasive developmental disorders, potentially impacting the results. This longitudinal study employed a clear exposure-outcome relationship, allowing for temporality to be ascertained and for an association to be examined.

## 5. Conclusions

This study examined the association between maternal ACEs and children’s sleep disruptions, considering independent and serial mediation through maternal depressive symptoms and children’s screen time. Findings indicate that the effects of mothers’ ACEs on their children’s sleep disruptions may act indirectly, with a particular emphasis on maternal depressive symptoms. Consistent with other research that did not observe direct effects of mothers’ ACEs on their children’s sleep quality more broadly [31,62], it is speculated that effects may operate entirely through indirect pathways. While screen time was posited to also serve as a mediator of the association, the null findings suggest that they may not help to explain the effects of mothers’ ACEs on children’s sleep quality, although additional research employing different measures of screen time is needed prior to drawing strong conclusions. Reducing depressive symptoms among mothers with a history of ACEs is a critical target for intervention to reduce children’s sleep disruptions and support healthier developmental pathways. Public health initiatives that focus on perinatal mental health screening and treatment, along with early parenting support, and an expansion of trauma-informed supports for mothers who have experienced ACEs, could produce intergenerational benefits. Additionally, addressing various social determinants of child health at both clinical and policy levels can be a key strategy to lessen the intergenerational impact of ACEs on child development and well-being.

## Figures and Tables

**Table 1:** Participant demographic characteristics (n = 622).

Variable	Number and Proportion of Participants (n, (%))	Mean (SD)	Missing Values (n, (%))
**Maternal Characteristics**			
*Age at Enrollment (Years)*	-	32.3 (4.0)	0 (0.0)
*Self-Identified Ethnicity*			4 (0.6)
White	550 (88.4)	-	
Chinese	41 (6.6)	-	
Latin American	11 (1.8)	-	
Black	8 (1.3)	-	
Other	6 (1.0)	-	
Indigenous	2 (0.3)	-	
*Education Level*			5 (0.8)
University Degree	305 (49.0)	-	
Post Graduate Degree	162 (26.0)	-	
Trade School	110 (17.7)	-	
High School or Less	40 (6.4)	-	
*Household Income*			11 (1.8)
>CAD 100k	361 (58.0)	-	
CAD 70k to CAD 99,999	160 (25.7)	-	
CAD 40k to CAD 69,999	69 (11.1)	-	
CAD 20k to CAD 39,999	16 (2.6)	-	
<CAD 10k	5 (0.8)	-	
**Child Characteristics**			
*Sex-Assigned-at-Birth*			0 (0.0)
Male	329 (52.9)	-	
Female	293 (47.1)	-	
*Gestational Age (Weeks)*	-	39.2 (1.7)	3 (0.5)
*Birthweight (Grams)*	-	3379.7 (522.1)	4 (0.6)

**Table 2:** Descriptive statistics for main study variables (n = 622).

Variable	Number and Proportion of Participants (n, %)	Mean (SD)	Missing Values (n, (%))
**Predictor Variable**			
*Mothers’ ACEs*	-	1.0 (1.5)	83 (13.3)
**Mediator Variables**			
*Mothers’ Depressive Symptoms*	-	4.8 (3.1)	3 (0.5)
*Child Screen Time (days/week > 1 h)*	-	3.7 (2.3)	111 (17.9)
	**Outcome Variable**		
*Child Wakes More Than Once*			0 (0.0)
Never (0 days/week)	447 (71.9%)	-	
Rarely (1 day/week)	138 (22.2%)	-	
Sometimes (2–4 days/week)	26 (4.2%)	-	
Usually (5–6 days/week)	9 (1.4%)	-	
Always (7 days/week)	2 (0.3%)	-	

## Data Availability

This study’s dataset is not publicly released, but it is available upon reasonable request to the corresponding author. The data are not publicly available due to ethical and privacy considerations.
